# Use of personal protective equipment by European Radiologists during the COVID-19 pandemic, a survey of the European Union of Medical Specialists (UEMS)

**DOI:** 10.1186/s13244-021-01154-8

**Published:** 2022-01-31

**Authors:** Miraude EAPM Adriaensen, Danoob Dalili, Hildo Lamb, Paolo Ricci

**Affiliations:** 1 UEMS Section of Radiology - Department of Medical Imaging, Zuyderland Medical Center, Henri Dunantstraat 5, 6419 PC Heerlen, the Netherlands; 2 Department of Radiology, Epsom and St Helier’s University Hospitals NHS Trust, Epsom, KT18 7EG Surrey UK; 3 UEMS Section of Radiology - Department of Radiology, Leiden University Medical Center, Albinusdreef 2, 2333 ZA Leiden, the Netherlands; 4 UEMS Section of Radiology - Department of Radiological, Oncological and Pathological Sciences, Policlinico Umberto 1, Sapienza University of Rome, Viale Regina Elena 324, 00168, Rome, Italy

**Keywords:** Ultrasound, Personal protective equipment, COVID-19

## Abstract

**Background:**

The current global pandemic of Coronavirus Disease 2019 (COVID-19) has profoundly impacted medical practitioners worldwide. This survey was formed by the Radiology Section of the European Union of Medical Specialists (UEMS) to establish the use of personal protective equipment (PPE) by European radiologists committed to providing face-to-face ultrasound services after the first few months of the COVID-19 global pandemic.

**Results:**

The results showed a heterogeneous picture within Europe regarding PPE used by European radiologists providing face-to-face ultrasound services. Ranging from full protection including full limb protection and double gloves to no PPE at all. In general, European radiologists were using more PPE when providing face-to-face ultrasound services in COVID-19 positive patients than in COVID-19 asymptomatic patients. In many member countries of the Radiology Section of the UEMS (19/30), there were no national guidelines with regard to the use of PPE by healthcare professionals committed to providing face-to-face ultrasound services.

**Conclusions:**

Our results showed that harmonization on a European level regarding the recommended use of PPE for European radiologists providing face-to-face ultrasound services is lacking. When the position statements and best practice recommendations on standards in ultrasound are revised, we recommend adding a paragraph on PPE.

## Key points

- Personal protective equipment for healthcare professionals providing face-to-face ultrasound services is recommended.
- In majority of countries, no national ultrasound personal protective equipment guidelines existed.
- When revising European ultrasound recommendations, add paragraph on personal protective equipment.

## Background

The current global pandemic of Coronavirus Disease 2019 (COVID-19) has profoundly impacted medical practitioners worldwide. In July 2020, Radiology Section of the European Union of Medical Specialists (UEMS), https://www.uemsradiology.eu/, was gravely concerned of conditions that some practitioners were facing now that ultrasound services were beginning to resume. Personal protection equipment (PPE) was a particular concern with many countries not able to safely conduct ultrasound examinations, putting front line practitioners at unnecessary risk [1]. Whilst we did not yet fully understand this disease in July 2020, we did know that Black, Asian and minority ethnic groups amongst medical practitioners and ancillary healthcare staff were amongst the highest mortality rates [2, 3].

This survey was formed by the Radiology Section of the UEMS to establish the use of PPE by European radiologists committed to providing face-to-face ultrasound services after the first few months of the COVID-19 global pandemic.

## Methods

### Study design

A questionnaire was developed collaboratively by representatives of the Radiology Section of the UEMS who approved international distribution of this survey in July 2020. In-line with previous studies, the free online web-based software “Google Forms” (Google LLC, Mountain View, CA, USA) was utilised to create and disseminate the survey and collect responses. In accordance with National Health Service (NHS) Health Research Authority criteria, this study did not require application for ethical approval [4]

The anonymised survey was composed of 31 questions; with the full list of questions and answers displayed in Table 1. Five questions required answers as multiple-choice (Likert or dichotomous) selections and the remaining twenty-six questions were ‘yes’ or ‘no’ questions. There were free text boxes available where elaboration to answers was invited. On July 22, 2020, invitations to participate in the survey were distributed via email to all representatives of the Radiology Section of the UEMS and its Divisions of Interventional Radiology and Neuroradiology. During the summer of 2020 two reminders were sent. Following a unanimous decision by the authors, the survey was concluded after two months in September 2020.

**Table 1:** Full list of questions included in the survey and answers from 46 respondents

Question	Answer
*Background information*	
1. Which country are you working in?	Austria: 2 (4.3%)
	Bulgaria: 2 (4.3%)
	Croatia: 1 (2.2%)
	Cyprus: 1 (2.2%)
	Czech Republic: 1 (2.2%)
	Denmark: 1 (2.2%)
	Estonia: 2 (4.3%)
	Finland: 1 (2.2%)
	France: 2 (4.3%)
	Greece: 1 (2.2%)
	Germany: 1 (2.2%)
	Hungary: 3 (6.5%)
	Iceland: 2 (4.3%)
	Ireland: 1 (2.2%)
	Italy: 2 (4.3%)
	Latvia: 1 (2.2%)
	Lithuania: 1 (2.2%)
	Luxembourg: 1 (2.2%)
	Malta: 1 (2.2%)
	Netherlands: 2 (4.3%)
	Norway: 1 (2.2%)
	Portugal: 1 (2.2%)
	Poland 2: (4.3%)
	Romania: 1 (2.2%)
	Slovenia: 1 (2.2%)
	Spain: 1 (2.2%)
	Sweden: 1 (2.2%)
	Switzerland: 2 (4.3%)
	Turkey: 3 (6.5%)
	United Kingdom: 4 (8.7%)
2. Which type of practice are you working in?	University Hospital: 34 (73.9%)
	Teaching Hospital: 6 (13%)
	General Hospital: 8 (17.4%)
	Private practice: 3 (6.5%)
	Elective outpatients: 1 (2.2%)
3. What types of ultrasound do you perform in your practice?	General ultrasound: 38 (82.6%)
	Musculoskeletal ultrasound: 20 (43.5%)
	Other: 19 (41.3%) Vascular: 4 (11%) Biopsy: 2 (4.3%) Neuro/Head and Neck: 2 (4.3%) Interventional: 4 (11%) Paediatric: 2 (4.3%) Breast: 1 (2.2%) None: 1 (2.2%)
*Dedicated PPE questions*	
In COVID-19 positive ( +) patients which PPE is used by patients?	
4. Masks	Non-surgical: 0 (0%)
	Surgical: 25 (54.3%)
	FFP2: 10 (21.7%)
	FFP3: 9 (19.6%)
	No facemask used: 2 (4.3%)
5. Goggles	Yes: 13 (28.3%)
	No: 33 (71.7%)
6. Face shield	Yes: 17 (37%)
	No: 29 (63%)
7. Gown	Yes: 24 (52.2%)
	No: 22 (47.8%)
8. Gloves	Yes: 24 (52.2%)
	No: 22 (47.8%)
9. Plastic screen/wall	Yes: 7 (15.2%)
	No: 39 (84.8%)
In COVID-19 positive ( +) patients which PPE is used by Doctors?	
10. Masks	Non-surgical: 0 (0%)
	Surgical: 12 (26.1%)
	FFP2: 18 (39.1%)
	FFP3: 16 (34.8%)
	No face mask used: 0 (0%)
11. Goggles	Yes: 37 (80.4%)
	No: 9 (19.6%)
12. Face shield	Yes: 38 (82.6%)
	No: 8 (17.4%)
13. Gown	Yes: 44 (95.7%)
	No: 2 (4.3%)
14. Gloves	Yes: 45 (97.8%)
	No: 1 (2.2%)
15. Plastic screen/wall	Yes: 9 (19.6%)
	No: 37 (80.4%)
16. Regarding COVID-19 asymptomatic patients, how is this determined?	PCR test: 18 (39.1%)
	Questionnaire by phone: 13 (28.3%)
	Questionnaire at the door of the hospital/practice: 29 (63%)
	We do not test/enquire about asymptomatic patients: 10 (21.7%)
	In case of positive questionnaire PCR test necessary: 3 (6.5%)
	All the patients undergoing an US exam should carry a non-surgical mask: 2 (4.3%)
	Check list when entering hospital including temperature testing: 1 (2.2%)
	No: 1 (2.2%)
17. Regarding COVID-19 asymptomatic patients, how many days in advance are they tested/questioned?	0–1 day: 9
	2–3 days: 6
	> 3 days: 1
In COVID-19 asymptomatic patients which PPE is used?	
18. Masks	Non-surgical: 1 (2.2%)
	Surgical: 33 (71.7%)
	FFP2: 4 (8.7%)
	FFP3: 4 (8.7%)
	No facemasks used: 4 (8.7%)
19. Goggles	Yes: 7 (15.2%)
	No: 39 (84.8%)
20. Face shield	Yes: 9 (19.6%)
	No: 37 (80.4%)
21. Gown	Yes: 15 (32.6%)
	No: 31 (67.4%)
22. Gloves	Yes: 18 (39.1%)
	No: 28 (60.9%)
23. Plastic screen/wall	Yes: 5 (10.9%)
	No: 41 (89.1%)
In COVID-19 asymptomatic patients which PPE is used by Doctors?	
24. Masks	Non-surgical: 0 (0%)
	Surgical: 29 (63%)
	FFP2: 9 (19.6%)
	FFP3: 6 (13%)
	No facemasks: 2 (4.3%)
25. Goggles	Yes: 21 (45.7%)
	No: 25 (54.3%)
26. Face shield	Yes: 20 (43.5%)
	No: 26 (56.5%)
27. Gown	Yes: 27 (58.7%)
	No:19 (41.3%)
28. Gloves	Yes: 39 (84.8%)
	No: 7 (15.2%)
29. Plastic screen/wall	Yes: 5 (10.9%)
	No: 41 (89.1%)
*Rules and guidelines*	
30. Do you have different rules for different types of ultrasound?	Yes: 13 (28.3%)
	No: 33 (71.7%)
31. Do you have national guidelines?	Yes:17 (37%)
	No: 29 (63%)

### Data analysis

Data were collected and tabulated independently via Google Forms (Google Forms, Google LLC, CA). Additionally, all responses were collected in an electronic spreadsheet (Microsoft Excel, Microsoft, Redmond, VA). The results were analysed by two researchers who have been previously involved in survey studies among other things performed by the ACI (Accreditation Council in Imaging), the ESSR (European Society of Musculoskeletal Radiology), and the BSSR (British Society of Skeletal Radiology). Descriptive statistics were used to summarise multiple-choice responses, with results expressed as number of respondents and percentages. A narrative analysis was conducted on the free text question to identify recurring themes.

## Results

**Fig. 1:**
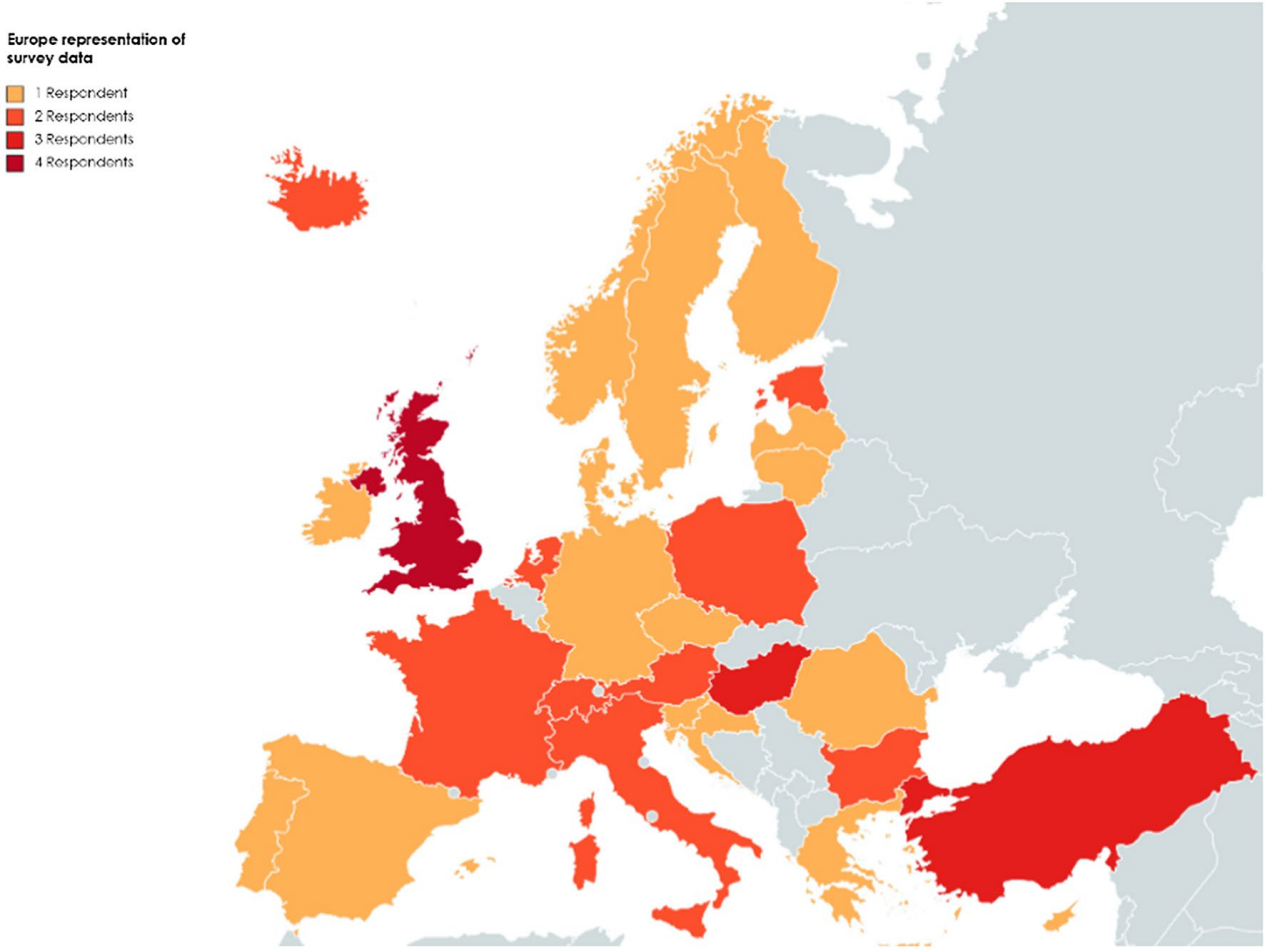
Distribution of completed surveys by country

The survey was distributed among all 31 member countries of the Radiology Section of the UEMS and its Divisions. Answers were received from 46 participants representing UEMS delegates from 30 European countries out of 31 member countries (97%) (Fig. 1). The full list of answers from all 46 respondents are presented in Table 1. The absolute number of completed answers, stratified by country is reported in Table 2.

**Table 2:** The distribution of completed surveys stratified by country

Which country are you working in?	Austria: 2 (4.3%)
	Bulgaria: 2 (4.3%)
	Croatia: 1 (2.2%)
	Cyprus: 1 (2.2%)
	Czech Republic: 1 (2.2%)
	Denmark: 1 (2.2%)
	Estonia: 2 (4.3%)
	Finland: 1 (2.2%)
	France: 2 (4.3%)
	Greece: 1 (2.2%)
	Germany: 1 (2.2%)
	Hungary: 3 (6.5%)
	Iceland: 2 (4.3%)
	Ireland: 1 (2.2%)
	Italy: 2 (4.3%)
	Latvia: 1 (2.2%)
	Lithuania: 1 (2.2%)
	Luxembourg: 1 (2.2%)
	Malta: 1 (2.2%)
	Netherlands: 2 (4.3%)
	Norway: 1 (2.2%)
	Portugal: 1 (2.2%)
	Poland: 2 (4.3%)
	Romania: 1 (2.2%)
	Slovenia: 1 (2.2%)
	Spain: 1 (2.2%)
	Sweden: 1 (2.2%)
	Switzerland: 2 (4.3%)
	Turkey: 3 (6.5%)
	United Kingdom: 4 (8.7%)

In total, 74% (34/46) of answers were received from members who practiced in university hospitals, 13% (6/46) had worked in teaching hospitals and 17% (8/46) had performed ultrasounds in general hospitals. A further 7% (3/46) had been exposed to ultrasound in private practice and 2% (1/46) in an elective outpatient clinic.

**Fig. 2:**
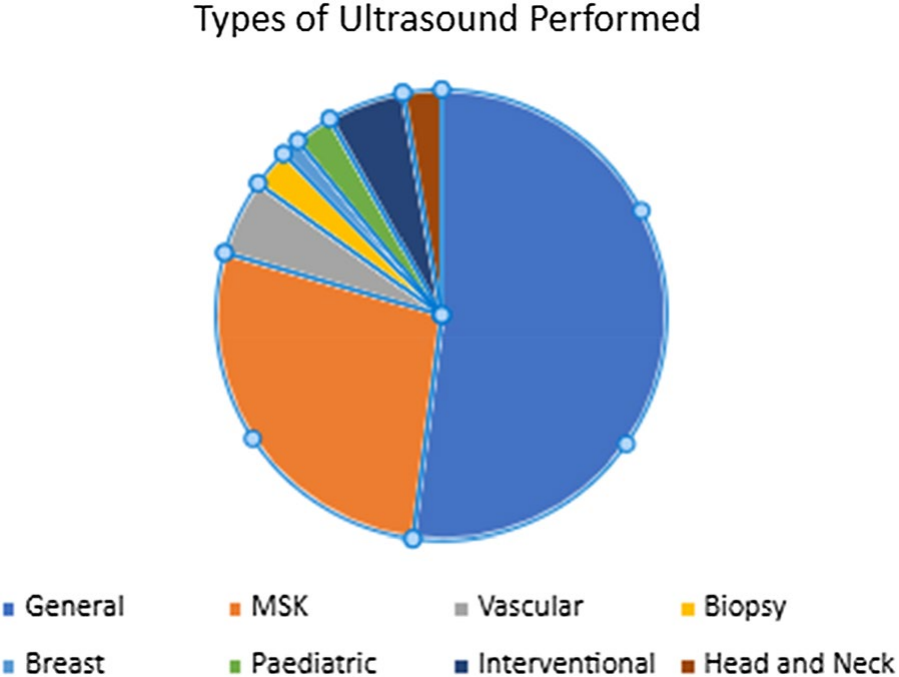
Types of ultrasound performed. MSK = musculoskeletal

The majority (83%; 38/46) of participants practiced general ultrasound, with just under a half (43%; 20/46) performed musculoskeletal ultrasound (Fig. 2). A number of participants also expressed performing other ultrasound examinations such as vascular (11%; 4/46), interventional (11%; 4/46) and paediatric (4%; 2/46) (Fig. 2).

With regard to dedicated PPE being worn by COVID-19 positive patients, 54% (25/46) respondents reported that patients wore surgical masks to appointments, whilst a further 22% (10/46) and 20% (9/46) of participants reported patients as wearing Filtering Facepiece (FFP)2 and FFP3 masks, respectively. 4% (2/46) respondents stated that COVID-19 positive patients did not wear facemasks in their department. With respect to goggles, 72% (33/46) of respondents stated that COVID-19 positive patients did not wear goggles in the department and a majority of 63% (29/36) reported face shields not being worn. 52% (24/46) of responses stated that patients wore gowns and the same number was also seen for gloves (52%; 24/46). Very few (15%; 7/46) respondents reported the use of a plastic screen/wall when scanning COVID-19 positive patients.

Where patients have been found to be COVID-19 positive, respondents reported that all radiologists wore masks with 26% (12/46) wearing surgical masks, 39% (18/46) wearing FFP2 masks and 35% (16/46) wearing FFP3 masks. Furthermore, the majority of respondents reported that radiologists used goggles (80%; 37/46), face shields (83%; 38/46), gowns (96%; 44/46) and gloves (98%; 45/46). The use of plastic screens still remained low with only 20% (9/46) of respondents reporting their use. In the free-text portion of the question one respondent expressed that in their department, full limb protection and double gloves were used as a precaution.

With respect to determining whether asymptomatic patients were COVID-19 negative, 63% (29/46) of respondents stated that a questionnaire was held at the door of the hospital/practice prior to the appointment. A further 28% (13/46) stated questionnaires were conducted by phone and 7% (3/46) would have a PCR test if the questionnaire is positive. 39% (18/46) of respondents indicated that their department performed initial polymerase chain reaction (PCR) tests to determine if asymptomatic patients were COVID-19 positive. No testing or enquiry of asymptomatic patients was reported by 22% (10/46) of respondents. Of the members that responded to the question, the majority (56%; 9/16) stated that testing/questioning was performed 0–1 days before an appointment.

Regarding COVID-19 asymptomatic patients, the vast majority of respondents (72%; 33/46) indicated that patients wore surgical masks, whilst four respondents (9%; 4/46) stated use of FFP2 and FFP3 alike. Alongside this 9% (4/46) of participants stated that asymptomatic patients wore no masks at all in their department. With respect to goggles and face shield usage, 85% (39/46) and 80% (37/46) of participants, respectively, reported no use of these protective measures by asymptomatic patients. A further 89% (41/46) of respondents stated that COVID-19 negative patients did not use plastic screens/walls at their scans. With regards to gown use in presumed COVID-19 negative patients, 67% (31/46) of survey participants reported no use of these by patients. A further 61% (28/46) reported patients not wearing gloves.

The results showed that in cases where patients were COVID-19 asymptomatic, 4% (2/46) of participants stated that doctors did not wear a face mask at all during appointments. The remaining responses stated that surgical face masks were the predominant type of mask used at 63% (29/46) whilst 20% (9/46) of respondents reported FFP2 use and 13% (6/46) FFP3. Just under half of respondents (46%; 21/46) stated that clinicians wore goggles whilst scanning COVID-19 asymptomatic patients. Use of face shields and gowns worn by clinicians whilst scanning COVID-19 asymptomatic patients were reported by 44% (20/46) of respondents and 59% (27/46), respectively. In COVID-19 asymptomatic patients, the use of gloves and plastic screens remained similar to that in COVID-19 positive patients with a high (85%; 39/46) usage of gloves and a low (11%; 5/46) usage of plastic screens/walls.

Of note is some mentioned in the free text that some healthcare workers in the department would use no PPE at all when scanning COVID-19 asymptomatic patients and in most departments US intervention required a negative PCR test to go ahead.

In the summer of 2020, 11 out of 30 countries (37%) reported to have national guidelines with regard to PPE when performing face-to-face ultrasound services. One respondent described how guidelines and recommendations on how to behave during encounters with patients that have or might have or had COVID-19 changed all the time during the pandemic. So the biggest challenge has been to keep up with the recommendations, on a weekly or even daily basis. Supplies of masks, gloves, ultrasound covers etcetera also changed very rapidly.

## Discussion

This survey was distributed in the summer of 2020 by the Radiology Section of the UEMS to establish the use of PPE by European radiologists committed to providing face-to-face ultrasound services after the first few months of the COVID-19 global pandemic hitting Europe. The results showed a heterogeneous picture within Europe with regard to PPE used by European radiologists providing face-to-face ultrasound services. Ranging from full protection including full limb protection and double gloves to no PPE at all. In general European radiologists were using more PPE when providing face-to-face ultrasound services in COVID-19 positive patients than in COVID-19 asymptomatic patients. In most countries, i.e. 19 out of 30 countries, there were no national guidelines with regard to the use of PPE by healthcare professionals committed to providing face-to-face ultrasound services.

One of the limitations of this survey inherent to the study design and distribution of the survey was the limited absolute number of radiologists who answered the survey, i.e. 46. However, as we received responses from 30 out of 31 member countries, we believe this survey gave a good representation of the situation of the use of PPE by European radiologists committed to providing face-to-face ultrasound services in the summer of 2020 after the first few months of the COVID-19 global pandemic.

Another limitation of this survey to keep the number of questions asked manageable to answer, was that the survey did not cover all the different situations and circumstances in Europe in which healthcare professionals were providing face-to-face ultrasound services. For example, instead of only COVID-19 positive patients and COVID-19 asymptomatic patients, there were also PCR-proven COVID-19 negative patients. Furthermore, PPE practices might have been different in an outpatient setting, an intra-hospital setting and on dedicated COVID-19 wards or dedicated COVID-19 hospitals.

Two important papers with regard to hygiene and infection prevention in ultrasound have been published by the European Society of Radiology Ultrasound Subcommittee (former  Ultrasound Working Group) [5, 6]. However, none of the papers has mentioned the use of personal protective equipment for neither the healthcare professional providing the face-to-face ultrasound services nor the patient [7].

As already mentioned by many European healthcare professional organisations in March 2020 and stated in the British Medical Journal (BMJ)’s proper PPE campaign, we repeat that all healthcare on the frontline should be given appropriate level of PPE of sufficient quality and quantity for each clinical setting to keep and make them feel safe [8, 9]. Therefore, we recommend adding a paragraph on personal protective equipment for health professionals providing face-to-face ultrasound services to the best practice recommendations on ultrasound when the position statements of the European Society of Radiology Ultrasound Subcommittee are revised [5, 6].

## Conclusions

To conclude, there is room for harmonisation on a European level with regard to the recommended use of personal protective equipment for healthcare professionals providing face-to-face ultrasound services. When the position statements and best practice recommendations on standards in ultrasound are revised, we recommend to add a paragraph on personal protective equipment.

## Data Availability

The dataset used and analysed is available from the corresponding author on reasonable request.

## Abbreviations

ACI: Accreditation Council of Imaging
BMJ: British Medical Journal
BSSR: British Society of Skeletal Radiologists
COVID-19: Coronavirus Disease 2019
ESSR: European Society of Musculoskeletal Radiology
FFP: Filtering Facepiece
NHS: National Health Service
PCR: Polymerase Chain Reaction
PPE: Personal Protection Equipment
SARS-CoV-2: Severe Acute Respiratory Syndrome Coronavirus 2
UEMS: European Union of Medical Specialists
